# Placental Expression of NEMO Protein in Normal Pregnancy and Preeclampsia

**DOI:** 10.1155/2019/8418379

**Published:** 2019-01-02

**Authors:** Agata Sakowicz, Michalina Lisowska, Lidia Biesiada, Elżbieta Płuciennik, Agnieszka Gach, Magda Rybak-Krzyszkowska, Hubert Huras, Bartosz Sakowicz, Hanna Romanowicz, Agnieszka W. Piastowska-Ciesielska, Mariusz Grzesiak, Tadeusz Pietrucha

**Affiliations:** ^1^Department of Medical Biotechnology, Medical University of Lodz, Zeligowskiego 7/9, Lodz, Poland; ^2^Department of Obstetrics, Perinatology, and Gynecology, Polish Mother's Memorial Hospital-Research Institute in Lodz, Rzgowska 281/289, Lodz, Poland; ^3^Department of Molecular Cancerogenesis, Medical University of Lodz, Zeligowskiego 7/9, Lodz, Poland; ^4^Department of Genetics, Polish Mother's Memorial Hospital-Research Institute in Lodz, Rzgowska 281/289, Lodz, Poland; ^5^Department of Obstetrics and Perinatology, University Hospital in Krakow, Kopernika 36, Krakow, Poland; ^6^Department of Microelectronics and Computer Science, Lodz University of Technology, Wolczanska 221/223, Lodz, Poland; ^7^Department of Clinical Pathomorphology, Polish Mother's Memorial Hospital-Research Institute in Lodz, Rzgowska 281/289, Lodz, Poland; ^8^Department of Comparative Endocrinology, Laboratory of Cell Cultures and Genomic Analysis, Medical University of Lodz, Zeligowskiego 7/9, Lodz, Poland

## Abstract

**Background:**

Preeclamptic pregnancies often present an intensified inflammatory state associated with the nuclear activity of NF*κ*B. NEMO is an essential regulator of nuclear factor kappa B (NF*κ*B) in cytoplasmic and nuclear cellular compartments. The aim of the present study is to examine the level and localization of the NEMO protein in preeclamptic and nonpreeclamptic placentas.

**Methods:**

The study includes 97 preeclamptic cases and 88 controls. NEMO distribution was analyzed immunohistochemically. Its localization in the nuclear and cytoplasmic fractions, as well as in total homogenates of placental samples, was studied by western blot and ELISA.

**Results:**

The western blot and ELISA results indicate a significant difference in NEMO concentration in the total and nuclear fractions between preeclamptic and control samples (*p* < 0.01 and *p* < 0.001, respectively). In the cytoplasmic complement, similar levels of NEMO were found in preeclamptic and control placentas. In addition, immunohistochemical staining revealed that the NEMO protein is mainly localized in the syncytiotrophoblast layer, with controls demonstrating a stronger reaction with NEMO antibodies. This study also shows that the placental level of NEMO depends on the sex of the fetus.

**Conclusions:**

The depletion of the NEMO protein in the cellular compartments of placental samples may activate one of the molecular pathways influencing the development of preeclampsia, especially in pregnancies with a female fetus. A reduction of the NEMO protein in the nuclear fraction of preeclamptic placentas may intensify the inflammatory state characteristic for preeclampsia and increase the level of apoptosis and necrosis within preeclamptic placentas.

## 1. Introduction

Preeclampsia is a multisystem, placentally mediated disorder which affects 5–8% of all pregnancies worldwide [1]. This phenomenon usually appears after week 20 of gestation and is characterized by elevated blood pressure (over 140/90 mmHg) usually accompanied by proteinuria (>300 mg for 24 hours or at least 2+ on a dipstick). However, the American College of Obstetricians and Gynecologists and the International Society for the Study of Hypertension in Pregnancy regard the occurrence of a new onset of hypertension combined with a new onset of thrombocytopenia, renal insufficiency, impairment of liver function, or neurological complications, together with the absence of proteinuria, as sufficient diagnostic criteria to recognize preeclampsia [2, 3].

Although our understanding of the risk factors and predictive markers for preeclampsia development is growing, the precise etiology of this phenomenon remains largely unknown. Numerous studies have pointed out that one of the main causes of preeclampsia is the incorrect implantation of the embryo into the uterine wall during the early stage of gestation. Abnormal, shallow placentation results in placental hypoperfusion, leading to hypoxia and oxidative stress and placental cell death [4]. This increases trophoblast turnover and elevates the level of fetal-free DNA and trophoblast-derived vesicles and debris in the blood of expecting women, thus inducing the hallmarks of preeclampsia: an inflammatory vascular response and endothelial damage to maternal vessels [5, 6].

NF*κ*B is one of the factors linked with the abundance of preeclamptic placental apoptosis [7]. A higher level of NF*κ*B gene expression has been observed in the placentas of preeclamptic mothers [8]. Moreover, increases in NF*κ*B gene expression account for the placental level of the NF*κ*B protein, especially subunits p65 and p50 [7, 9, 10].

One of the main activators of NF*κ*B on the classical pathway is the IKBKG protein (known as NEMO or IKK*γ*). As our previous study has pointed out, the level of *NEMO* gene expression was significantly higher in the maternal and fetal blood of preeclamptic cases than in healthy controls, but lower in the placentas of pregnancies complicated by hypertension and proteinuria [11]. NEMO deficiency and disruption has been found to result in spontaneous cell death by apoptosis or necroptosis; however, this also triggers the expression of proinflammatory mediators in adjacent cells with normal NEMO levels [12–15]. This has been confirmed both on animal models and *in vitro* studies on human kidney embryotic cells (HEK-293 NEMO-null cells) [12, 13, 16]. In addition, experiments on the cardiomyocytes of male NEMO^Hko^ mice demonstrate that NEMO inactivation in the heart leads to spontaneous, progressive impairment of cardiac function manifested by extensive fibrosis and remodeling; this leads to hypertrophy and heart failure attributable to an oxidative stress-mediated mechanism and myocyte cell death [17, 18].

The changes taking place in preeclamptic placentas have also been observed in experimental models of NEMO-deficient cells and tissues [19]. These changes may occur in response to the disruption of both NF*κ*B-related and NF*κ*B-independent processes where NEMO plays a critical role. Certainly, NEMO interacts with over one hundred other proteins in cells; these include more than 30 kinases and almost 25 proteins involved in signal transductions [20]. In addition, the action of NEMO is not restricted to the cytoplasm. In the nucleus, it plays significant roles as a repressor of NF*κ*B-dependent gene expression and as a stabilizer of factors related to multiple cellular processes including proliferation, differentiation, or apoptosis [21–24].

Following on from these previous observations about the dualistic role of the NEMO protein in the cell and our previous results related to the depletion of *NEMO* gene expression in placentas complicated by preeclampsia, the present study examines whether the level of the NEMO protein and its localization in the placental cells may be associated with the occurrence of preeclampsia. It also determines whether the time of appearance of symptoms of disease (early or late preeclampsia) or the sex of the fetus may be associated with the level of the NEMO protein in placental cells.

## 2. Methods

### 2.1. Patient Selection and Data Collection

In total, 97 preeclamptic cases and 88 controls were recruited to the study. Written informed consent was obtained from all women, and the study protocol was approved by the Medical University of Lodz Ethical Committee.

The study population included singleton pregnancies complicated by preeclampsia, which was diagnosed according the following criteria: hypertension (systolic blood pressure over 140 mmHg and diastolic blood pressure over 90 mmHg; measured twice with an interval of at least six hours) and proteinuria (above 300 mg/24 hours or at least 2+ on a dipstick). Both symptoms appeared after twenty weeks of gestational age in previously normotensive women. All women qualified to the study group delivered by caesarean section. Control voluntaries were healthy women with singleton normotensive pregnancies.

The further following exclusion criteria were used for both the preeclamptic and control groups: no chronic diseases, maternal BMI before pregnancy <30 kg/m^2^, and no fetal chromosomal abnormalities. To eliminate any potential influence associated with the form of delivery, placentas in the control group were obtained only from women delivered by caesarean section without prior contraction. The caesarean section in the control group was conducted due to the following criteria: transverse or breech position of the fetus, ophthalmological indications, orthopedic indications, and an increased risk of uterine rupture due to a previously performed caesarean section.

Placental fragments about 2 cm^3^ were taken immediately after birth. The fragments were trimmed approximately 5 cm from the site of the umbilical cord insertion into the placenta. The decidua and amnion were removed. Following the drainage of excess blood, the placental samples were immediately washed in sterile phosphate buffered saline (PBS) and set in RNA*later* (Ambion) to protect RNA and protein from degradation. Tissues destined for protein isolation were stored at −20°C, and the protein fractions were isolated within two years from the collection date. The protein was stored at −80°C for further analyses.

### 2.2. Cytoplasmic and Nuclear Subfraction Isolation

The cytoplasmic and nuclear subfractions were isolated using a commercial kit (Nuclear/Cytosol Extraction Kit, BioVision Inc., USA) with some modification. The concentration of the isolated protein was assessed by fluorescence with the use of a NanoOrange™ Protein Quantification Kit (Invitrogen). The clearance of the isolated subfraction was confirmed by western blot analyses with an anti-GAPDH antibody for the cytoplasmic subfraction and anti-Lamin A/C for the nuclear subfraction. The isolated proteins were stored at −80°C for further analyses.

### 2.3. ELISA Method

NEMO protein concentration was measured in the following placenta subfractions using a sandwich ELISA kit (Human IKBKG ELISA Kit, Wuhan Fine Biotech Co., China): total homogenates of placental samples, cytoplasmic protein, and nuclear protein. Total homogenates were prepared according to the manufacturer's instructions with slight modifications. Ten-fold diluted samples and appropriate dilutions of calibrators were added in 100 *μ*l amounts to microplate wells precoated with an antibody specific for the human NEMO protein. Each sample was assayed in duplicate. Following a 90-minute incubation at 37°C, the microplate was washed and incubated with a biotin-labeled antibody against the human NEMO protein. After another one-hour incubation, the microplate was washed and incubated for 30 minutes with HRP-streptavidin conjugate (SABC). In the next step, the microplate was washed and incubated in a substrate solution to detect peroxidase activity, yielding a blue color in its presence. This blue solution became yellow after the addition of the stop solution. The absorbance was measured at 450 nm. The concentration of the NEMO protein in the studied samples was calculated on the basis of nanograms (ng) of NEMO protein per 100 *μ*g of total protein isolated in the sample.

The results were assessed according to a calibration curve. The intra-assay coefficient of variation (CV) was below 3% for both the nuclear and whole homogenate fractions, and it was 3.8% for the cytoplasmic fraction. The interassay CV values were below 9%, 12%, and 13% for nuclear, cytoplasmic, and total protein fractions, respectively; these are regarded as acceptable by FDA recommendations [25].

### 2.4. Western Blot Analyses

Around 10–15 nuclear subfractions from each of the control, early, and late preeclamptic placenta groups were pooled randomly within each group. The same procedures were used for the cytoplasmic subfraction and for total homogenates. The protein concentration was measured by fluorescence. The samples were loaded onto a 10% SDS-PAGE gel and separated under reducing conditions. Following this, they were transferred to a PVDF microporous membrane (Merck Millipore, Billerica, MA). In the next step, the membrane was incubated with 5% nonfat milk in TBST buffer (250 mM Tris-HCL pH 7.6, 1.5 M NaCl, 0.01% Tween) for one hour at room temperature, and then overnight at 4°C with a primary antibody diluted in 1% nonfat milk in TBST buffer: 1 : 500 for IKBKG (MA5-15155, Thermo Scientific), 1 : 500 for Lamin A/C (MA3-1000, Thermo Scientific), and 1 : 1000 for GAPDH (MA5-15738, Thermo Scientific). Following incubation, the membrane was washed in TBST buffer, incubated for one hour at room temperature with labeled alkaline phosphatase (A3562, Sigma-Aldrich; dilution 1 : 10000 in 1% nonfat milk in TBST) secondary antibody, and again washed. The analysis was then performed using an ECL detection kit according to the manufacturer's instructions. The optical density of bands was analyzed using the ImageJ software [26].

### 2.5. Immunohistochemistry

For histological studies, full-depth tissue samples were placed in 10% neutral buffered formalin solution for 24–48 hours. After incubation, fragments of the placentas were dehydrated through a graded series of ethanol and embedded in paraffin. Serial sections (5 mm) were placed on a slide for immunohistochemical analyses and stained using an automated IHC/ISH staining instrument (BenchMark XT, Ventana). CC1 tris buffer with a slightly basic pH was added for 90 minutes to retrieve the antigen. The tissue was then incubated for 32 minutes with rabbit polyclonal anti-IKBKG antibodies (Thermo Scientific) diluted 1 : 100 in antibody diluent (Ventana). The antigen-primary antibody complex was incubated with the enzyme-labeled secondary antibody and visualized with a hydrogen peroxide substrate and 3,3′-diaminobenzidine tetrahydrochloride (DAB) chromogen. The slides were then examined under a light microscope (Olympus). The optical density (OD) of the immunohistochemical slides used for NEMO protein analysis in preeclamptic and control placentas was analysed by the use of Olympus Microimage™ Image Analysis software, version 4.0 for Windows (Olympus Optical Co. GmbH), as described elsewhere [27].

### 2.6. Statistical Analyses

Statistical analyses were performed using Statistica version 13.1 (StatSoft). The normal distribution of continuous data was checked with the Shapiro–Wilk test. Normally distributed data was further analyzed with Student's *t*-test, while nonnormally distributed data was analyzed with the Mann–Whitney *U* test. The categorical variables were evaluated with the chi-square test or with Yates's correction. The NEMO levels in the early preeclampsia, late preeclampsia, and control samples were compared using the Kruskal–Wallis test with the post hoc Dunn test (for ELISA results) and using the ANOVA with the post hoc Tukey tests (for western blot results) for the total homogenates, cytoplasmic fractions, and nuclear fractions. A *p* value below 0.05 for the test results was considered statistically significant.

## 3. Results

The clinical and laboratory characteristics, as well as the perinatal outcomes of all patients qualified to the study and control groups, are presented in Table 1.

No significant difference was observed between the study and control groups for maternal age, number of miscarriages, or hematological parameters, except platelet count. The distribution of fetal sex varied between groups, although not significantly. Maternal BMI was slightly higher in the study group, whereas the newborns born from preeclamptic pregnancies were significantly lighter and taller. The number of primiparous cases dominated in the study group.

### 3.1. ELISA Test and NEMO Protein

**Figure 1:**
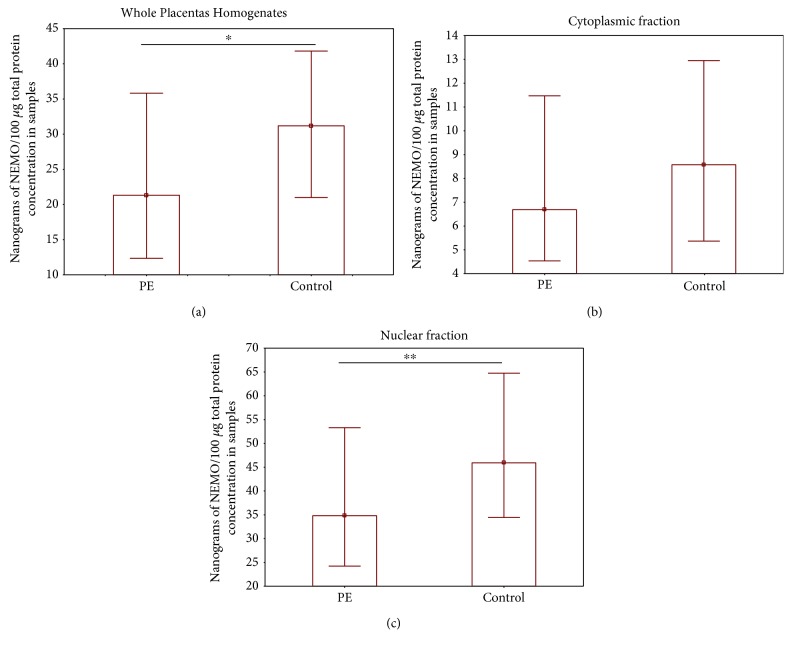
The comparison of the levels of the NEMO protein in the preeclamptic (PE) and control placental samples. (a) Whole placenta homogenates, (b) cytoplasmic fraction, and (c) nuclear fraction. Data are presented as a median and lower-upper quartile. The data were analyzed with the Mann–Whitney *U* test. Asterisks indicate significant differences (^∗^*p* < 0.001 and ^∗∗^*p* < 0.01) compared to control.

The level of the NEMO protein was found to be significantly lower in preeclamptic placentas compared to controls in both the total homogenates and nuclear fraction. Although a low level of the NEMO protein was also observed in the cytoplasmic fraction, the results were not statistically significant. The NEMO protein concentration revealed by ELISA (ng NEMO per 100 *μ*g total protein) is given in Figures 1(a)–1(c).

**Figure 2:**
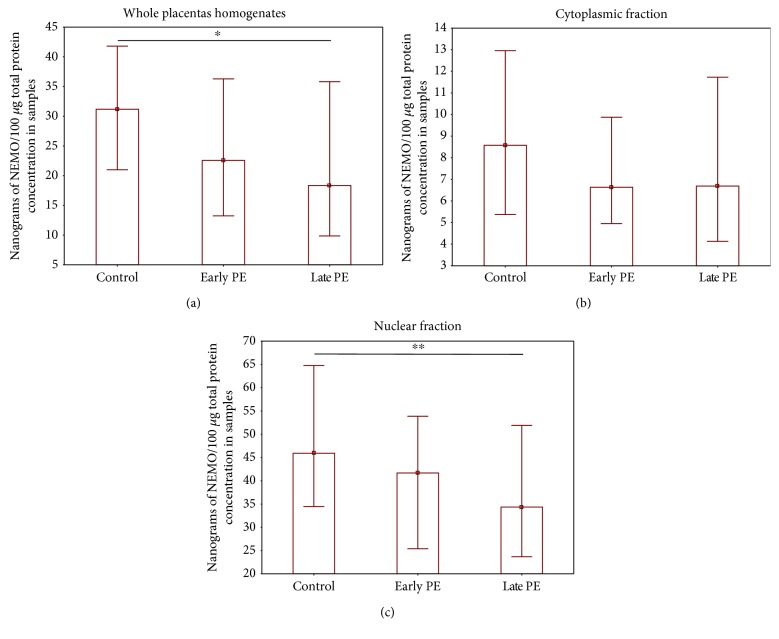
Comparison of the NEMO protein concentrations (ng NEMO per 100 *μ*g total protein) between early and late preeclamptic subgroups and controls. (a) Whole placenta homogenates, (b) cytoplasmic fraction, and (c) nuclear fraction. Data are presented as median and interquartile range; *p* value was calculated by the Kruskal–Wallis test with the post hoc Dunn test. Asterisks indicate significant differences (^∗^*p* < 0.002 and ^∗∗^*p* < 0.022) between the late preeclampsia group and control group.

After dividing the study group into early (<34 week of pregnancy) and late preeclamptic (≥34 week of gestation) subgroups, it was observed that only the late preeclamptic group differed significantly from controls with regard to NEMO protein level. No discrepancies in the level of the NEMO protein were observed between early and late preeclampsia, or between early preeclampsia and control (Figures 2(a)–2(c)).

**Figure 3:**
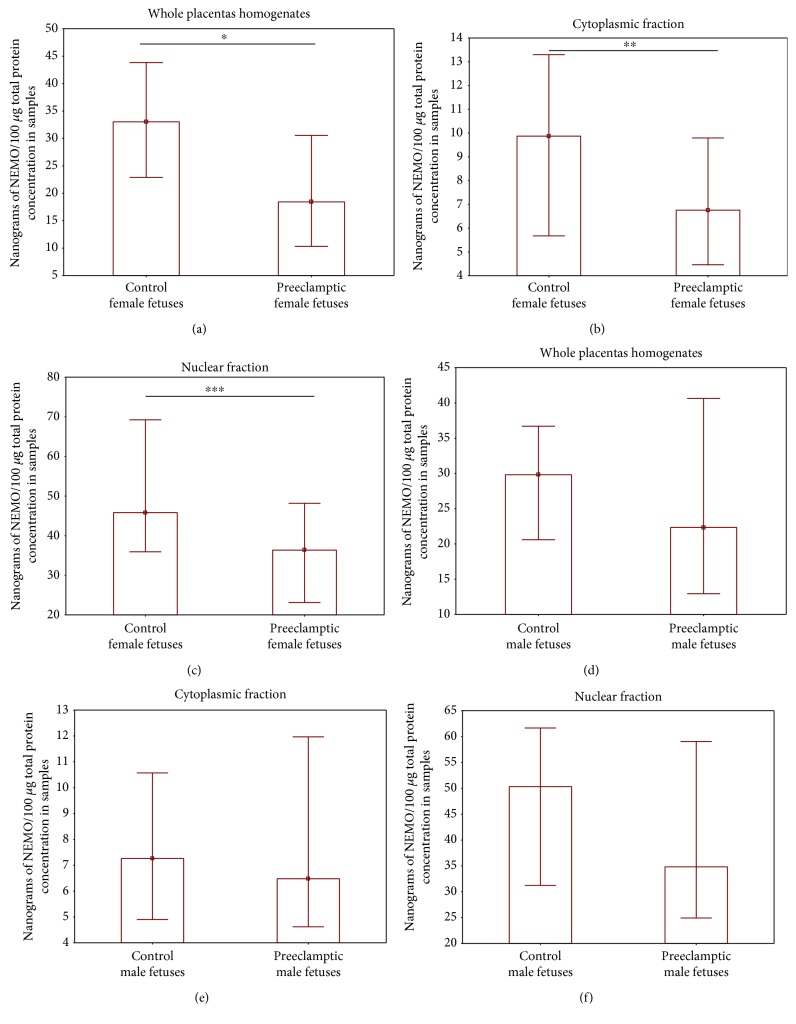
A comparison of NEMO protein levels between the study and control groups in female and male placentas. (a, b, c) Results for female placentas for whole and cytoplasmic and nuclear fractions, respectively. (d, e, f) Results for male placentas for whole and cytoplasmic and nuclear fractions, respectively. Data are presented as median and interquartile range. Data were analyzed by the Mann–Whitney *U* test. Asterisks indicate significant differences (^∗^*p* < 0.004, ^∗∗^*p* < 0.024 and ^∗∗∗^*p* < 0.013) in comparison to control.

Interestingly, the level of the placental NEMO protein depends on the sex of the fetus. Although the NEMO protein level between male and female placentas did not differ significantly, it was observed that placentas of male fetuses (study and control together) present a lower level of NEMO both in cytoplasmic and nuclear fractions than the placentas of all female fetuses qualified to the study (median 6.5 vs 8.3 for the cytoplasmic fraction, 36 vs 42 for the nuclear fraction). The whole, cytoplasmic fraction, and nuclear fraction of preeclamptic placentas of female fetuses demonstrated a significant depletion in NEMO concentration compared to those of female controls (Figures 3(a)–3(c)). In contrast, the median NEMO protein concentrations in all fractions were insignificantly lower in the preeclamptic placentas of male fetuses than male controls (Figures 3(d)–3(f)).

### 3.2. Immunohistochemical and Western Blot Results

**Figure 4:**
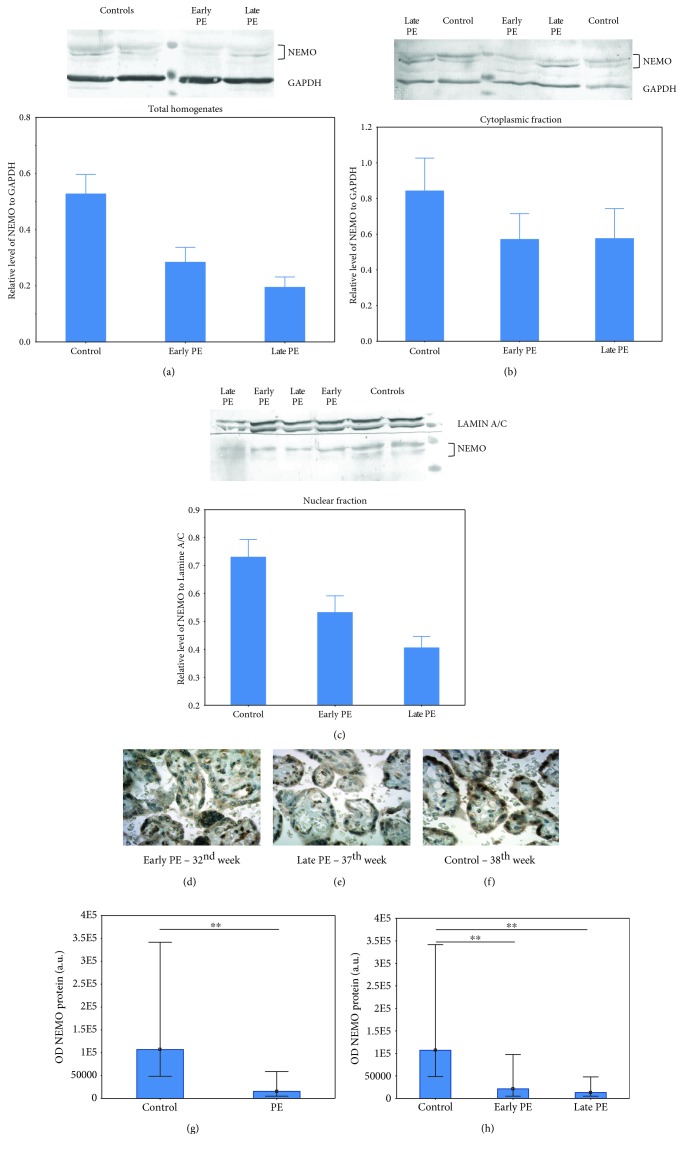
Western blot and immunohistochemical staining analyses of the NEMO protein in placentas from early, late preeclampsia, and noncomplicated pregnancies. Western blot analyses of the NEMO protein for total homogenates (a), cytoplasmic fraction (b), and nuclear fraction (c). The western blot analyses are presented as mean ± SEM (*n* = 16 for each subfraction). Asterisks indicate significant differences (^∗^*p* value < 0.05) between late preeclampsia and control. Immunostaining of NEMO in early preeclampsia (d), late preeclampsia (e), and control placentas (f). The histograms (g, h) represent the median value and interquartile range of the optical density (OD) of the NEMO protein between whole preeclamptic and control groups (g) and between the early preeclamptic group, late preeclamptic group, and control (h). The OD density is given on an arbitrary scale in arbitrary units (a.u.). Asterisks indicate significant differences (^∗∗^*p* < 0.001).

The western blot analyses of the randomly merged samples of total homogenates, cytoplasmic fraction, and nuclear fraction (*n* = 16 for each subfraction) found that the level of the NEMO protein is significantly depleted in total homogenates and nuclear fractions of preeclamptic placental samples (*p* < 0.01) but not in the cytoplasmic fraction (*p* = 0.202). However, after the separation of the preeclamptic group into early and late preeclampsia, the nuclear fraction was found to be significantly depleted only in the late preeclampsia compared to controls (*p* = 0.004). Similarly, the level of the NEMO protein in total homogenates was found only in late preeclamptic subgroups compared to controls (*p* = 0.016, ANOVA post hoc Tukey's test) (Figures 4(a)–4(c)).

Five placental samples were chosen randomly from each of the early preeclampsia, late preeclampsia, and control groups and examined using immunohistochemical techniques. The results indicate that the NEMO protein is mainly localized in the syncytiotrophoblast (Figures 4(d)–4(f)). The syncytiotrophoblast cells from normotensive gestations demonstrated a stronger reaction for the NEMO protein in comparison to early and late preeclampsia (Figures 4(g) and 4(h)).

## 4. Discussion

NEMO is an essential component of the IKK complex, playing a significant role in the activation of NF*κ*B through the classical (canonical) pathway. Although NEMO does not have any kinase activity, it is believed that it may serve as a scaffolding protein organizing the formation of the IKK*α* and IKK*β* complex in the cytoplasm. Moreover, NEMO may schedule between the cytoplasmic and nuclear compartment, where it may play a dual role in the cellular regulation of processes related to NF*κ*B. While the NEMO protein is perceived as a regulator of NF*κ*B activity along the canonical pathway in the cytoplasm, it also competes with NF*κ*B and IKK*α* for binding with CBP protein and represses the transcriptional action of NF*κ*B in the nucleus [24, 28].

The results of the western blotting and ELISA testing in the present study revealed a low level of the NEMO protein in the total homogenates of preeclamptic placental samples compared to controls. Nuclear localization of NEMO was also significantly lower in the study samples (*p* < 0.01). This depletion in NEMO in preeclamptic placentas was confirmed by the immunohistochemical analysis. The optical density histograms revealed significantly weaker staining for the NEMO protein in the syncytiotrophoblast layer of the preeclamptic placental villous in comparison to controls (Figures 4(g) and 4(h)) (*p* < 0.001).

Such depletion in the level of NEMO, a natural nuclear repressor of the transcriptional activity of NF*κ*B, may be one of the reasons for the development of preeclampsia. Immunohistochemical studies indicate the presence of an elevated level of NF*κ*B in preeclamptic placentas, especially in the syncytiotrophoblast layer, where NF*κ*B is mainly localized in the nucleus [7, 10]. The localization of NF*κ*B in the nuclear fraction alters the expression of many genes [10], with the higher expression of most of these genes being linked with the pathogenesis of preeclampsia. Increased NF*κ*B activity in the nucleus accounts for the elevated placental levels of IL-1, Il-8, TNF*α*, cytosolic phospholipase A2, and thromboxane in preeclamptic pregnancies [10, 29–33].

Moreover, NF*κ*B is perceived as a regulator of the genes implicated in the apoptosis, necrosis, and necroptosis (programmed necrosis) pathways [34, 35]. All of these processes are more pronounced in preeclamptic placentas [30, 35]. NEMO has also been found to regulate the process of necrosis independently of NF*κ*B; in addition, NF*κ*B-independent cooperation by NEMO with other proteins may activate one of the possible death pathways in the placental cells and promote the development of preeclampsia. A study conducted on the Jurkat cell line found that NEMO binds to the ubiquitinated form of RIP1 [36], an essential factor implicated in the control of necrosis and necroptosis [36–38]. The interaction between NEMO and the ubiquitinated form of RIP1 prevents RIP1 from playing a role in the necrotic death pathway [36]. NEMO has also been found to prevent cell death in NEMO^IEC-KO^ mice [14]. Moreover, NEMO may protect cells from apoptosis by its negative effect on the interaction between RIP1 and CASPASE-8 [39].

The human protein microarray study found that NEMO binds to almost 112 different proteins, allowing it to regulate a wide range of cell signaling pathways. This group of NEMO-binding proteins includes a large number of signaling kinases and proteins regulating the cell cycle, only some of which are implicated in the NF-*κ*B signaling pathway [20]. Moreover, Fenner et al. [20] reported that over 32% of the kinases that interact with NEMO are localized in cytoplasm/nuclear fractions, whereas 22% are reserved only for the nuclear compartment [20]. In the nucleus, a direct interaction has been observed between NEMO and the c-Myc protein, leading to c-Myc stabilization and phosphorylation and to overexpression of genes regulated by c-Myc [22]. This human oncogene has been implicated in the progression of human placenta development [40] by influencing the regulation of genes essential for vasculogenesis and angiogenesis, such as VEGF [41]. Therefore, a reduction of the NEMO protein in the nuclear fraction of preeclamptic placentas may be responsible for the disturbances observed in the concentration of c-Myc and VEGF. Both proteins are depleted in preeclamptic pregnancies [42–45]. Moreover, it is thought that NEMO is involved in the recruitment of histone deacetylases (HDACs), these being enzymes which modify the acetylation level of histone and nonhistone protein and regulate the accessibility of transcription factors to the DNA in the nucleus [24, 46]. It is thought that NEMO deficiency may significantly disturb the process of HDAC recruitment. Numerous studies indicate that deacetylases play a significant role in angiogenesis. The inhibition of these enzymes inhibits angiogenesis by influencing the expression of proangiogenic and angiogenic factors [47]. Estela et al. [48] report that the inhibition of HDAC activity by TSA (trichostatin A) leads to the reduction of trophoblast invasion by reducing metalloproteinase gene expression and increasing that of their inhibitors [48]. Inadequate trophoblast invasion and vascular remodeling are the main reasons of preeclampsia development [49]. Additionally, histone deacetylase inhibition promotes chymase activation (called a chymotrypsin-like protease) and increases the production of angiotensin II in cell cultures [50]. Higher levels of both enzymes have been implicated in the pathomechanism of preeclampsia [51, 52]. Interestingly, the inhibition of histone deacetylases also significantly influences the elevation of gene expression levels regulated by NF*κ*B [24]: HDAC proteins target NF*κ*B to repress the expression of its regulated genes [53].

**Figure 5:**
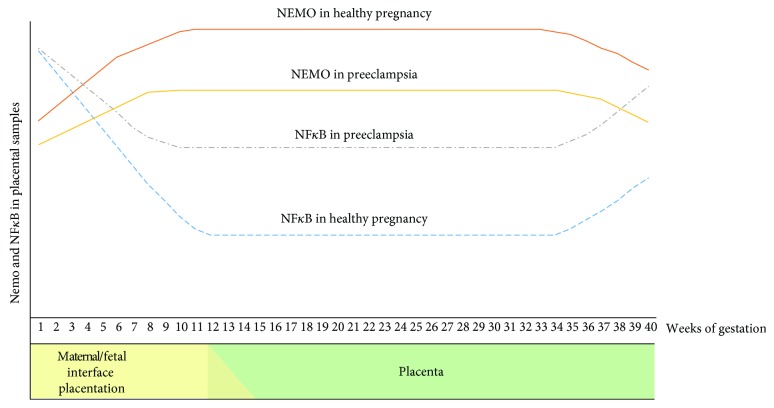
Relationship between the nuclear fraction of NEMO and NF*κ*B in healthy and preeclamptic pregnancies. Red line—hypothetical changes in the NEMO protein level during gestation without any complications. Yellow line—hypothetical changes in the NEMO protein level during gestation complicated by preeclampsia. Blue line—changes in the NF*κ*B protein level during gestation without any complications. Grey line—changes in the NF*κ*B protein level during gestation complicated by preeclampsia. At the beginning of pregnancy, the activity of NF*κ*B is high, and the nuclear level of the NEMO protein may be low, to open the implantation window. The transcriptional activity of NF*κ*B decreases over the course of a pregnancy to allow it to continue, in the same time the NEMO content of the nuclear fraction should be high to additionally repress the NF*κ*B activity in the nucleus. In the third trimester of noncomplicated pregnancy, the nuclear activity of NF*κ*B again increases to prepare the maternal organism for delivery, therefore the NEMO protein level in the nucleus should decrease.

Our findings suggest that the level of the NEMO protein depends on gestational age. Significantly lower levels of NEMO in total homogenates and nuclear fractions were identified in the placentas of women developing preeclampsia after the 34^th^ week of pregnancy compared to controls. The multiple comparison test found no significant difference in the level of the NEMO protein between early and late preeclampsia and between early preeclampsia and controls. Hence, the nuclear level of the NEMO protein appears to change during the course of pregnancy to maintain the appropriate transcriptional activity of NF*κ*B. The beginning of pregnancy may be characterized by high NF*κ*B activity and low nuclear NEMO level to open the implantation window and to develop the communication network between the mother and fetus [54]; however, the transcriptional activity of NF*κ*B then decreases over the course of pregnancy to allow it to continue [54]. It is possible that in noncomplicated pregnancies, the level of NEMO in the nucleus increases during this stage. As the transcriptional activity of NF*κ*B in the nucleuses of placentas complicated by preeclampsia is high, the NEMO content of the nuclear fraction should be low. In the third trimester of pregnancy, the nuclear activity of NF*κ*B again increases to evoke the inflammatory state and to prepare the maternal organism for delivery [54]. Therefore, in noncomplicated pregnancies, the NEMO protein level should decrease. As the level of NF*κ*B is much higher in preeclamptic pregnancies than in noncomplicated pregnancies in the last trimester, the level of the NEMO protein in the nuclear fraction should be much lower in the complicated pregnancies (Figure 5).

The literature indicates that preeclampsia is characterized by depletions in the levels of progesterone and estrogen [55]; the two hormones both repress NF*κ*B and are repressed by it. Progesterone and estrogens are believed to be involved in the inhibition of NF*κ*B transcriptional activity [56, 57], thus facilitating the immunosuppressive state needed to maintain pregnancy [56]. Some studies suggest that sexual dimorphism may exist between gestations with male and female fetuses, with regard to the expression of cytokines, testosterone, and other gonadal steroids [9, 58]. Steroid hormones regulate the expression of *NEMO*; for example, the estrogen receptor binds to the promoter region of *NEMO*. Elsarraj et al. [59] found that the level of NEMO increased both in biopsy samples of patients suffering from intraductal carcinoma and in breast cancer cell lines upon estrogen and progesterone treatment. The significant difference in the NEMO concentration between female preeclamptic placentas and controls observed in the present study, as well as the lack of a significant relationship between placental NEMO level and the development of preeclampsia in women bearing a male fetus, suggests that the pathomechanism of preeclampsia may be slightly divergent based upon fetal sex. Moreover, this observation may also suggest that NEMO may be implicated in the regulation of one of the pathways affecting preeclampsia. Further studies should be conducted to determine the regulation of NEMO concentration regarding fetal sex and preeclampsia development.

## 5. Conclusions

The present study demonstrates the relationship between the NEMO protein level and the occurrence of preeclampsia. A low level of the NEMO protein in the nucleus of trophoblast cells may induce one of the molecular pathways leading to preeclampsia development. It may induce dysregulation of NF*κ*B transcriptional activity, which may explain why numerous studies have reported high NF*κ*B activity and elevated levels of inflammatory markers in preeclamptic pregnancies. Moreover, as NEMO regulates necrotic and apoptotic pathways independently of NF*κ*B, the presence of a NEMO deficiency in preeclamptic placentas increases the chance of intensified apoptosis and necrosis. Additionally, further studies performed over the course of gestation are needed to confirm the role of NEMO in the development of preeclampsia and to determine whether assessment of the NEMO protein may be a good predictive marker for the outcome of preeclampsia.

## Figures and Tables

**Table 1:** Clinical, hematological, and pregnancy characteristics of the study and control groups.

Parameter	Study group (*n* = 97) Mean ± standard deviation	Control group (*n* = 88) Mean ± standard deviation	*p* ^(1)^

WBC (10^3^/*μ*l)	10.4 ± 2.4	10.6 ± 2.2	0.560
RBC (10^6^/*μ*l)	4.1 ± 0.48	4.2 ± 0.41	0.376
HB (g/dl)	12.4 ± 2.3	12.4 ± 1.1	0.987
HCT (%)	35 ± 3.5	36.2 ± 2.6	0.099
MCV (fl)	85 ± 6.3	87 ± 5.2	0.072
MCHC (g/dl)	34 ± 2.4	34 ± 1.6	0.051
PLT (10^3^/*μ*l)	203 ± 60	222 ± 55	0.041
BMI (kg/m^2^)	26 ± 3	25 ± 4	0.001
Newborn weight (g)	2371 ± 956	3401 ± 445	<0.001
Newborn high (cm)	48 ± 7	54 ± 3	<0.001
Maternal age at the time of delivery (years)	31 ± 6	32 ± 4	0.135
Week of delivery (week)	31 ± 2 (early preeclampsia)	39 ± 1	<0.001
	38 ± 2 (late preeclampsia)		0.001

Parameter	Study group *n* (%)	Control group *n* (%)	*p* ^(2)^
Primiparous	66 (68%)	35 (40%)	<0.001
Miscarriage	16 (16%)	15 (17%)	0.920
Male sex of the fetus	58 (60%)	40 (45%)	0.051

Legend: BMI, body mass index; WBC, white blood cells; RBC, red blood cells; HB, hemoglobin concentration; HCT, hematocrit; MCV, mean corpuscular volume; MCHC, mean corpuscular hemoglobin concentration; PLT, platelets; kg/m^2^, kilograms/meter square; *μ*l, microliter; g/dl, grams/deciliter; %, percent; fl, femtoliter; g, grams; cm, centimeter; *n*, number of cases; *p*^(1)^—*p* value calculated by Student's *t*-test; *p*^(2)^—*p* value calculated by chi^2^ or chi^2^ with Yates's correction tests.

## Data Availability

The data used to support the findings of this study are included within the article.
